# Long non-coding RNAs as biomarkers and therapeutic targets for ischemic stroke

**DOI:** 10.1016/j.ncrna.2022.09.004

**Published:** 2022-09-10

**Authors:** Galina Sufianova, Alina Shumadalova, Yao Wenhao, Ilgiz Gareev

**Affiliations:** a Рeoples’ Friendship University of Russia (RUDN University), 6 Miklukho-Maklaya Street, Moscow, 117198, Russian Federation; b Bashkir State Medical University, Ufa, Republic of Bashkortostan, 450008, Russia; c Department of Pharmacology, Tyumen State Medical University, 54 Odesskaya Street, 625023, Tyumen, Russia; d The First Affiliated Hospital of Harbin Medical University, Harbin, 150001, China

**Keywords:** Long non-coding RNAs, Ischemic stroke, Pathogenesis, Therapeutic, Biomarkers, Risk factors, Angiogenesis, Neurogenesis

## Abstract

**Background:**

The problem of ischemic stroke (IS) has become increasingly important in recent years, as it ranks first in the structure of disability and mortality, crowding out other vascular diseases. In this regard, the study of this pathology and the search for new therapeutic and diagnostic tools remains an urgent problem of modern medical science and practice. Long non-coding RNAs (lncRNAs)-based therapeutics and diagnostic tools offer a very attractive area of study. Therefore, this systematic review aims at summarizing current knowledge on promising lncRNAs as biomarkers and therapeutic targets for IS exploring original articles and literature reviews on *in vivo*, in vitro and *ex vivo* experiments.

**Methods:**

The current systematic review was performed according to PRISMA guidelines. PubMed, MEDLINE and Google Scholar databases were comprehensively explored to perform the article search.

**Results:**

34 eligible studies were included and analyzed: 25 focused on lncRNAs-based therapeutics and 9 on lncRNAs-based diagnosis. We found 31 different lncRNAs tested as potential therapeutic and diagnostic molecules in cells and animal model experiments. Among all founded lncRNA-based therapeutics and non-invasive diagnostic tools, nuclear enriched abundant transcript 1 (NEAT1) emerged to be the most investigated and proposed as a potential molecule for IS diagnosis and treatment.

**Conclusions:**

Our analysis provides a snapshot of the current scenario regarding the lncRNAs as therapeutic molecules and biomarkers in IS. Different lncRNAs are differently expressed in IS, and some of them can be further evaluated as therapeutic targets and biomarkers for early diagnosis and prognosis or treatment response. However, despite many efforts, none of the selected studies go beyond preclinical studies, and their translation into clinical practice seems to be very premature.

## 1 Introduction

Ischemic stroke (IS) is one of the leading causes of disability and death worldwide. The main cause of IS (regardless of the pathogenetic subtype) is a violation of the blood supply to the brain, causing a deficiency of oxygen and nutrients, leading to damage to the nervous tissue. Persistent neurological deficit after IS is largely a socio-economic burden for both the patient and society [1]. The currently used methods for the treatment and prevention of this pathology, despite significant progress, have not been effective enough, partly due to incomplete understanding of the molecular mechanisms involved in the pathogenesis of IS.

To date, a search is underway for new effective diagnostic methods and therapeutic agents for cerebrovascular diseases. Long non-coding RNAs (lncRNAs) are among the most intensively studied RNA molecules in recent times. LncRNAs are a class of noncoding RNAs with a length of more than 200 nucleotides that play a regulatory role in various biological processes of the cell, such as apoptosis, cell cycle, proliferation, cell differentiation, etc. [2]. An increasing number of studies demonstrate the direct role of lncRNA in the pathogenesis of various human diseases, including oncological, inflammatory, cardiovascular, etc. [3,4]. Of serious interest are studies that describe the role of lncRNA in the pathogenesis of IS, a complex, multifactorial pathology with significant etiological heterogeneity. The pathogenesis of IS includes (among other things) endothelial dysfunction and changes in the walls of cerebral vessels under the influence of arterial hypertension and atherosclerosis, which are known risk factors for the development of IS [5]. In arterial hypertension and atherosclerosis, aberrant lncRNA expression occurs due to the regulation of the expression of some miRNAs and target genes, where lncRNA is involved in such processes as phenotypic changes in vascular smooth muscle cells (VSMCs), inflammation, degradation of the extracellular matrix (ECM), endothelial dysfunction, death cells and the production of reactive oxygen species (ROS) [6,7]. Numerous pathological molecular processes are involved in the development of IS, including inflammation, dysfunction of the blood-brain barrier (BBB), cerebral edema, and neuronal death [8]. In this systematic review, we look at research related to lncRNA and AI and try to explain the complex relationship between them. The clinical potential of lnRNAs to develop new diagnostic and therapeutic strategies for IS will also be discussed.

## 2 Material and methods

### 2.1 Data sources and search strategy

We conducted a comprehensive search for original papers and literature reviews demonstrating the potential role of lncRNAs in the development of IS. Databases including PubMed, MEDLINE, and Google Scholar. The search included reports published before August 2022. Key words including "ischemic stroke", or "stroke", or "pathogenesis", or "diagnosis", or "treatment", or " molecular mechanisms" and "long non-coding RNAs", or "epigenetics", or "biomarker", or " therapeutic targets", or "signal pathways". In addition, the literature list of each relevant study was searched to identify other relevant papers.

### 2.2 Inclusion and exclusion criteria

To avoid heterogeneity in the selected articles, we applied inclusion and exclusion criteria. We focused on papers reporting on lncRNAs involved in the pathogenesis of IS and as diagnostic and therapeutic tools for IS; no previous systematic review has combined all of these lnRNAs. Studies were included that used both human and animal material to explore the potential role of lncRNA in IS. Studies that reported diseases similar to the pathogenesis of IS, such as hemorrhagic stroke, inflammatory brain diseases, and vascular malformations, were excluded. In addition, studies reporting lncRNA in transient and chronic ischemic stroke were excluded (Table 1).

**Table 1:** Inclusion and exclusion criteria.

Inclusion criteria	Exclusion criteria
English language	Publication type: meta-analysis, systematic review, conference abstract, case reports, personal communications, and letters to editor
Studies published between 2016 and 2022	Studies considering circulating long non-coding RNAs (lncRNAs) for diagnostic and prognostic tools testing, engineering, and validation
Studies considering long non-coding RNAs (lncRNAs) as therapeutic targets	Studies on the therapeutic use of long non-coding RNAs (lncRNAs) and diagnostic and prognostic use of circulating lncRNAs in patients with: • hemorrhagic stroke • inflammatory brain diseases • vascular malformations • chronic ischemic stroke

Studies using circulating long non-coding RNAs (lncRNAs) as diagnostic and prognostic biomarkers.

## 3 Results

### 3.1 Basic information of enrolled articles

**Fig. 1:**
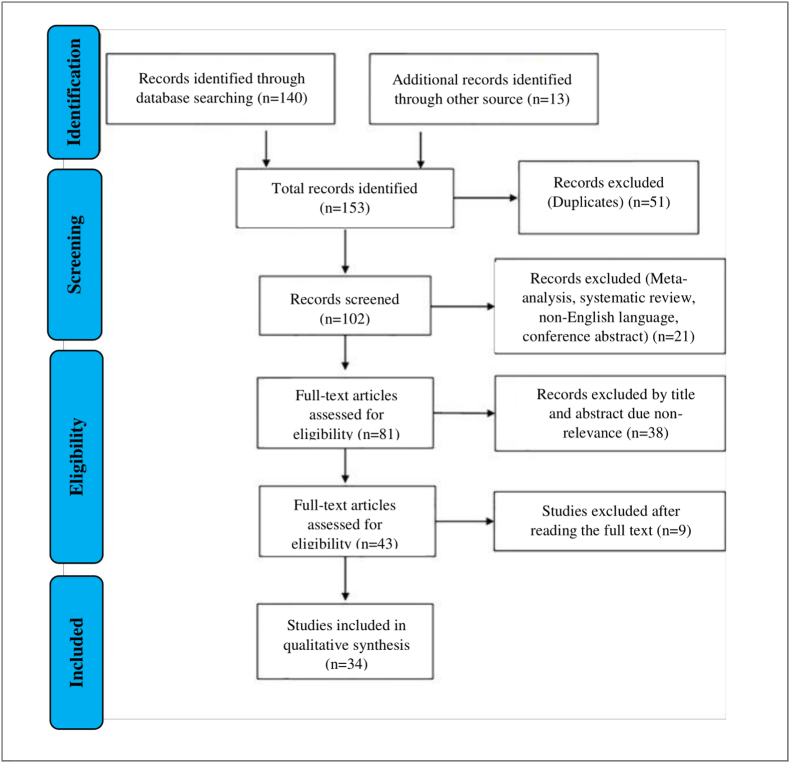
Flowchart for the strategy searches and selection processes.

A flowchart showing the publication search and detailed article selection process is shown in Fig. 1. The oldest studies presented in all the databases queried are from 2010. 51 duplicates were removed, of the remaining 102 entries, 21 were excluded as non-research articles or non-English literary publications. Of the remaining 81 articles, 38 were excluded due to irrelevance after checking the title and abstract. The remaining 34 eligible articles were downloaded and read, and 9 of them were excluded due to lack of information. The final 34 eligible articles were grouped according to two research areas: 1) lncRNAs as therapeutic targets in IS and 2) circulating lncRNAs as non-invasive biomarkers of IS. A total of 24 lncRNAs have been identified for therapeutic and diagnostic applications. In particular, 17 lncRNAs (MEG3, H19, NEAT1, SNHG1, HOTTIP, LOC102640519, Gm4419, MRAK048635_P1, MALAT1, AK094457, AF131217.1, ATB, 430945, LEF1-AS1, MALAT1, GAS5, and HIF1A-AS2) were accepted as potential therapeutic agents and 7 lncRNAs (NEAT1, ENST00000568297, ENST00000568243, NR_046084, ANRIL, ZFAS1, and MIAT) in as potential non-invasive biomarkers.

### 3.2 Analysis of studies on lncRNAs and risk factors of IS

It is no secret that arterial hypertension is a major risk factor for the development of IS and is one of the main components of the metabolic syndrome (obesity, dyslipidemia, and hyperglycemia/insulin resistance) [9]. In addition, arterial hypertension accompanied by atherosclerosis is a complex, multifactorial complex disease, and their joint development is determined by a combination of genetic predisposition and environmental factors [10]. The pathogenesis of arterial hypertension is based on dysregulation of the endothelium, VSMCs dysfunction, nitric oxide (NO) synthesis, increased oxidative stress, impaired angiogenesis, activation of the sympathetic nervous system, and changes in the activity of the renin-angiotensin-aldosterone system (RAAS) [[9], [10], [11]]. Based on the polyfunctionality of lncRNAs and their direct involvement in the pathogenesis of many diseases, it can be assumed that lncRNAs play one of the key roles in pathophysiological processes that contribute to the development of hypertension and atherosclerosis, both individually and in combination [12,13]. For example, growth arrest-specific long non-coding RNA 5 (GAS5) is one of the best studied lncRNAs involved in the pathogenesis of hypertension and vascular remodeling. LncRNA GAS5 expression has been found to be downregulated in the plasma of hypertensive patients and in the arteries and retina of spontaneously hypertensive rats. LncRNA GAS5 knockdown has been shown to result in VSMCs phenotype changes, vascular remodeling, and microvascular dysfunction. In addition, the proliferation, migration, and resistance to oxidative stress of human umbilical vein endothelial cells (HUVECs) and VSMCs were altered by GAS5 siRNA [14].

Another lncRNA studied in the pathogenesis of atherosclerosis is metastasis-associated lung adenocarcinoma transcript 1 (MALAT1). A recently published study suggested a direct role for MALAT1 in the development and progression of atherosclerosis and demonstrated that MALAT1 exhibits anti-inflammatory properties in part by inhibiting miR-503 expression *in vivo*. In particular, a decrease in MALAT1 activity in hematopoietic cells leads to increased formation of atherosclerotic lesions and inflammation in ApoE −/− mice fed a high fat diet (HFD). The progression of atherosclerotic lesions was due to an increase in the number of inflammatory cells in the bone marrow and increased adhesion in vitro and *in vivo*. In addition, enhanced adhesion of bone marrow cells was restored due to the inactivation of miR-503. Accordingly, MALAT1 expression in human atherosclerotic plaques was reduced compared to healthy vascular tissue and, moreover, MALAT1 expression was reduced in symptomatic patients compared to asymptomatic patients [15]. The mechanisms of lncRNA regulation in hypertension and atherosclerosis are summarized in Table 2 [[16], [17], [18], [19], [20], [21], [22]].

**Table 2:** Long non-coding RNAs (lncRNAs) involved in the pathogenesis of hypertension and atherosclerosis, with a presentation of the mechanisms of their regulation.

LncRNA	Disease	Expression	Target	Biological function	References
MRAK048635_P1	Hypertension	Down	Cyclin-dependent kinase 2 (CDK2) and cyclin-dependent kinase 4 (CDK4), cyclin D1 and cyclin E, caspase3, retinoblastoma protein (p-Rb), alpha-smooth muscle actin (α-SMA), poly(ADP-Ribose) polymerase 1 (PARP), and calponin	Causes a phenotypic change in vascular smooth muscle cells (VSMCs) from a contractile to a secretory phenotype. Promotes proliferation and migration of VSMCs and inhibits their apoptosis	[16]
Metastasis-associated lung adenocarcinoma transcript 1 (MALAT1)	Hypertension	Down	Notch homolog 1 (Notch-1)	Decreased relative expression of transcription factors associated with endothelial dysfunction, inflammation, and oxidative stress. Inhibition of endothelial cells (ECs) apoptosis	[17]
AK094457	Hypertension	Up	Peroxisome proliferator-activated receptor γ (PPARγ)	Enhances angiotensin II-induced hypertension and endothelial dysfunction	[18]
AF131217.1	Atherosclerosis	Down	miR-128–3p/Krüppel-like factor 4 (KLF4) axis	Reduced inflammation on the endothelial surface	[19]
LncRNA activated by TGF- β (ATB)	Atherosclerosis	Up	Transforming growth factor beta 1 (TGF-β1) and caspase-3	Apoptosis and inhibition of endothelial cells (ECs) proliferation	[20]
430945	Atherosclerosis	Up	Receptor tyrosine kinase like orphan receptor 2 (ROR2)/Ras homolog family member A (RhoA)	Promotes migration and proliferation of vascular smooth muscle cells (VSMCs)	[21]
LEF1 antisense RNA 1 (LEF1-AS1)	Atherosclerosis	Up	miR-544a/Phosphatase and tensin homolog deleted on chromosome 10 (PTEN) axis	Promotes proliferation and migration of vascular smooth muscle cells (VSMCs)	[22]

### 3.3 Analysis of studies on lncRNAs and IS

Hundreds of aberrantly expressed lnRNAs have been identified using methods such as real-time reverse transcription-PCR (qRT-PCR), microarray or next generation sequencing (NGS), in patients with IS, in vitro and *in vivo* [23]. In Table 3 systematically presents some lncRNAs that affect such fundamental processes of IS pathogenesis as cell death, BBB dysfunction, inflammation, and microglial activation [[24], [25], [26], [27], [28], [29], [30]].

**Table 3:** Long non-coding RNAs (lncRNAs) involved in ischemic stroke (IS) pathogenesis.

LncRNA	Process	Target	Expression	Study model	Biological function	References
Maternally expressed gene 3 (MEG3)	Apoptosis, necrosis and inflammation	miR-485/absent in melanoma 2 (AIM2) axis	Up	Middle cerebral artery occlusion (MCAO) model/Reperfusion (*in vivo*) and oxygen glucose deprivation (OGD) (in vitro)	Inhibits the death of neurons and reduces the area of infarction. Reduces the inflammatory process	[24]
H19	Apoptosis and necrosis	miR-19a/DNA-binding protein inhibitor ID-2 (Id2) axis	Up	Middle cerebral artery occlusion (MCAO) model/Reperfusion (*in vivo*) and oxygen glucose deprivation (OGD) (in vitro)	Enhances neuronal apoptosis and infarction	[25]
Nuclear enriched abundant transcript 1 (NEAT1)	Inflammation	Wnt/β-catenin signal pathway	Up	Oxygen glucose deprivation (OGD)/reperfusion (in vitro)	Microglial activation and stimulation of the inflammatory process	[26]
Small nucleolar RNA host gene 1 (SNHG1)	Blood-brain barrier dysfunction, cerebral edema and apoptosis	miR-338/hypoxia-inducible factor 1-alpha (HIF-1α) axis	Up	Oxygen glucose deprivation (OGD) (in vitro)	Increases the survival of endothelial (ECs) cells and inhibits their apoptosis. Reduces blood-brain barrier permeability and cerebral edema	[27]
HOXA transcript at the distal tip of antisense RNA (HOTTIP)	Apoptosis and carbohydrate metabolism	miR-143/hexokinase 2 axis	Up	Middle cerebral artery occlusion (MCAO) (*in vivo*) and oxygen glucose deprivation (OGD) (in vitro)	Increases the survival of neurons and suppresses their apoptosis. Promotes neuronal proliferation and stimulates glycolytic processes	[28]
LOC102640519	Blood-brain barrier dysfunction, cerebral edema and apoptosis	Homeobox protein (Hox-C13HOXC13), tight junction protein 1 (ZO-1) and vascular endothelial growth factor (VEGF)	Up	Middle cerebral artery occlusion (MCAO) (*in vivo*) and oxygen glucose deprivation (OGD)/reperfusion (in vitro)	Promotes an increase in the permeability of the blood-brain barrier and cerebral edema	[29]
Gm4419	Inflammation	Nuclear factor kappa B (NF-Κb), tumor necrosis factor-α (TNF-α), interleukin-1β (IL-1β), and interleukin-6 (IL-6)	Up	Oxygen glucose deprivation (OGD)/reperfusion	Microglial activation and stimulation of the inflammatory process	[30]

#### 3.3.1 LncRNAs and angiogenesis

Angiogenesis is the process of formation of new vessels from existing ones, and the process itself plays an important role in vascular remodeling and functional recovery after IS. It is known that angiogenesis is controlled by many key (angiogenic) factors, such as vascular endothelial growth factor (VEGF) [31]. Neovascularization in the brain parenchyma can cause an increase in cerebral blood flow, which ultimately increases the amount of oxygen and nutrients delivered to the ischemic area. The induction of angiogenesis by various therapeutic approaches that act on angiogenic factors seems to be a useful approach in the treatment of patients with IS [32]. The results of recent studies have shown that lncRNAs are important regulators of angiogenesis in cerebrovascular diseases [33]. VEGF is one of the most studied proangiogenic factors that plays an important role in angiogenesis, and its expression may be increased after IS [31]. The study of the mechanisms of regulation of VEGF activity after IS is important for the development of new targeted therapies. Lee et al., using a stroke model, namely by performing occlusion of the middle cerebral artery (MCAO) in rats, showed that miR-153–3p expression was reduced in the ischemic region of the brain, and hypoxia-inducible factor-1a (HIF - 1a) and its downstream targets (vascular endothelial growth factor A (VEGF-A) and Notch homolog 1 (Notch1)) were activated [34]. It has also been observed that hypoxia induces the expression of lncRNA HIF1A, antisense RNA 2 (HIF1A-AS2). And the final result was that HIF1A-AS2 promotes angiogenesis during hypoxia by activating the HIF-1a/VEGF-A/Notch1 signaling pathway by inhibiting miR-153–3p in HUVECs.

Zhan et al. found that the expression level of lncRNA maternally expressed gene 3 (MEG3) and NADPH oxidase 4 (NOX4) in cerebral vascular endothelial cells (ECs) is upregulated after oxygen-glucose deprivation/reperfusion *in vivo* [35]. It was noted that a decrease in MEG3 expression protects the endothelium of the microvasculature of cerebral vessels from induced oxygen-glucose deprivation/reperfusion of endotheliocytes apoptosis by reducing the expression of NOX4 and p53, as well as by reducing the level of intracellular reactive oxygen species (ROS). Decrease in MEG3 expression also increases HIF-1a and VEGF expression. In addition, p53 can stimulate NOX4 activity by direct binding to NOX4 promoters. This result indicates that MEG3 mediates post-IS angiogenesis through regulation of the p53/NOX4 axis.

#### 3.3.2 LncRNAs and neurogenesis

It is known that stimulation of neurogenesis occurs after IS as a protective reaction to injury. After IS neuronal progenitor cells can proliferate and migrate to the lesion [36]. Evidence suggests that IS-induced neurogenesis contributes to the functional recovery of IS patients. Using MCAO model, Wang et al. showed using immunofluorescence that downregulation of lncRNA H19 can reduce the area of diseased (ischemic) tissue and aid in the recovery of neurological damage (confirmed by the Rotarod and balancer tests) after IS [37]. Notch1 signaling has been reported to play an important role in the regulation of neurogenesis. The expression of Notch1 was also regulated by the transcription factor p53. To determine whether lncRNA H19 prevents neurogenesis through inactivation of the p53/Notch1 signaling pathway, the authors first attempted to elucidate the effect of lncRNA H19 on p53 activity under ischemic conditions. Using qRT-PCR, they proved that inhibition of lncRNA H19 expression can activate BCL2 associated X, apoptosis regulator (Bax) and the protein inhibitor of intracellular cyclin-dependent kinase 1A (CDKN1A), i.e., the p53 transcriptional activity complex. In other words, lncRNA H19 overexpression can inhibit p53 activity during IS. In addition, the results of Western blot showed that inhibition of lncRNA H19 can increase the expression level of p53. Notch1 expression was also upregulated by lncRNA H19 inhibition and attenuated by p53 inhibition based on a decrease in lncRNA H19 activity.

### 3.4 Analysis of studies on circulating lncRNAs as biomarkers

Instrumental diagnostics of IS at present based on neuroimaging modalities [1]. Given that these tests may not be available, accurate and reliable analysis of blood markers can help in early diagnosis (prevention), real-time diagnosis and prognosis of patients with IS. In contrast to acute coronary syndrome, for which there are many specific plasma or serum biomarkers (e.g. troponin) used both to diagnose and assess the severity of myocardial infarction, there are no established non-invasive markers for IS patients [38]. Most biomarkers associated with IS and proposed for diagnosis and prognosis are proteins such as inflammatory markers (e.g. C-reactive protein), S100β protein and D-dimer, matrix metalloproteinase-9 (MMP-9) [39]. In many biological fluids of the human body (whole blood, plasma/serum, or cerebrospinal fluid), numerous lncRNAs, called circulating lncRNAs, have been found [40]. Circulating lncRNAs can be secreted from cells into human biological fluids as part of extracellular carriers (exosomes and microvesicles (MVs)) or as part of apoptotic bodies and lipoproteins [41]. Such lncRNAs are resistant to RNases and show specificity for a particular pathology, which makes them attractive as new non-invasive diagnostic and prognostic biomarkers.

Circulating lncRNAs have been repeatedly investigated as diagnostic and prognostic biomarkers in various human diseases, including cerebrovascular diseases (Table 4) [[42], [43], [44], [45], [46], [47]]. It has been shown that circulating lncRNAs can be new potential biomarkers in IS for several reasons: 1) non-invasive detection method; 2) high stability in human fluids (such as blood); 3) are measured in many other body fluids; 4) are highly sensitive to disease; 5) can be detected in the early stages of IS, while protein markers are found in the circulation only when a significant amount of tissue damage has already occurred; 6) play a role in almost all cellular functions; 7) promising for rapid and accurate diagnosis of IS subtypes; and 8) are less complex molecules than most biological molecules in the blood, making analysis easier [[48], [49], [50]].

**Table 4:** The value of circulating long non-coding RNAs (lncRNAs) as non-invasive biomarkers for ischemic stroke (IS) diagnosis and prognosis.

lncRNA	Sample	Regulation	Diagnostic value	Prognostic value	Specificity, %	Sensitivity, %	Area under the ROC curve (AUC) value	Reference
Nuclear enriched abundant transcript 1 (NEAT1)	Plasma	Up	Yes	Yes	82.9	64.3	0.80	[42]
ENST00000568297, ENST00000568243 and NR_046084	Peripheral whole blood	Up	Yes	No	80.0 (combined)	82.8 (combined)	0.84 (combined)	[43]
ANRIL	Plasma	Down	Yes	No	71.2	72.2	0.76	[44]
ZNFX1 antisense RNA 1 (ZFAS1)	Leukocytes (WBC)	Down	Yes	No	48,6	89,3	0,727	[45]
Myocardial infarction associated transcript (MIAT)	Leukocytes (WBC)	Up	Yes	Yes	80,4	74,1	0,84	[46]
Antisense non-coding RNA in the INK4 locus (ANRIL)	Serum	Up	Yes	No	83,7	70,1	0,85	[47]

## 4 Discussion

The development and progression of vascular diseases, including IS, can be associated with both activation and a decrease in the expression of lncRNAs in cells. Therefore, approaches to gene therapy aimed at activating or suppressing the expression of specific lncRNAs for IS are currently being actively developed. Ways to increase or decrease lncRNAs expression include delivery of lncRNAs by viral vectors (lentiviruses) or non-viral vectors (inorganic or organic nanoparticles) [51]. The use of antisense oligonucleotides, transcriptional repression, and gene editing can be used to suppress lncRNA expression. Despite intensive research on the role of lncRNA in cerebrovascular diseases, there are currently no lncRNA-based therapeutics in this area that are applicable in clinical trials. There are several limitations to the development of therapy with the use of lncRNA in IS: 1) low efficiency of delivery to the vasculature and brain, as well as the likely need for repeated delivery; 2) the functions and mechanisms through which lncRNA influence the pathogenesis of IS are much more complex and diverse than those of other non-coding RNAs like miRNAs; 3) most lncRNAs that are localized in the cell nucleus act as epigenetic regulators; and 4) there are problems with the delivery of lncRNA, which can be overcome by chemical functionalization of the surface of nanoparticles, targeting specific ligands overexpressed by cells in the vessel wall or in brain cells in response to corresponding pathological stimuli [52,53]. In addition, most lncRNAs lack conservation between species, limiting the usefulness of preclinical animal studies. One possible strategy to overcome these problems is to identify direct target genes associated with the pathogenesis of IS (for example, using NGS) and use in vitro and *in vivo* preclinical studies to assess the potential role of these genes in the pathogenesis of IS [54]. Given more intensive research followed by clinical trials in patients, the use of lncRNA in IS therapy in modern clinical practice may become a reality.

One of the main obstacles in the development of specific biomarkers and effective therapeutic agents for diseases of the central nervous system (CNS) is the BBB. The BBB is a complex structure that controls the supply of nutrients and oxygen from the bloodstream to the CNS and prevents the accumulation of neurotoxins [55]. But at the same time, the BBB allows the passage of cationic or small fat-soluble molecules with a molecular weight of up to 400 kDa. Such transporters transport glucose and amino acids, while higher molecular weight molecules (insulin and transferrin) cross the BBB through receptor-mediated endocytosis [56]. However, the BBB is believed to be responsible for preventing the release of molecules specific for CNS diseases (e.g., tumors) into the bloodstream [57]. Current data indicate that the BBB is not an obstacle to the passage of lncRNA from the CNS into the bloodstream. It is known that, under pathological conditions, circulating lncRNAs can enter the bloodstream from the CNS through the BBB, which makes them potential indicators of CNS diseases, including IS. On the other hand, there are very few data on the transition of circulating lncRNA from the blood to the brain tissue. It is known that circulating small interfering RNAs with a molecular mass of 14 kDa, like lncRNAs, cannot diffuse through the BBB [58].

## 5 Conclusion

In recent years, progress has been made in uncovering the potential role of lncRNA in the pathogenesis of IS. LncRNAs may contribute to the progression of IS by regulating the activation certain target genes or signaling pathways, leading to the activation of microglia, increased inflammation, cell death, and impaired BBB function. On the contrary, there are lncRNAs that promote functional recovery by enhancing neurogenesis, angiogenesis and neuroprotection. Compared to studies examining the role of miRNAs in the pathogenesis of IS, the role of lncRNAs in the development of IS remains largely unknown. Further research is likely to discover new lncRNAs and their targets, which will allow a better understanding of the pathophysiological mechanisms underlying IS. Animal studies in MCAO and in vitro oxygen-glucose deprivation/reperfusion models will continue to be useful in determining the role of lncRNA in the pathogenesis of IS. Search for new lncRNAs and elucidation of their functions and mechanisms in IS will help in the development of non-invasive biomarkers for diagnosis and prognosis, as well as therapeutic agents in IS.

## Funding

This study was supported by the Bashkir State Medical University Strategic Academic Leadership program (PRIORITY-2030).

## Declaration of competing interest

The authors declare no conflict of interest, financial or otherwise.
